# Predicting Antibody Affinity Changes upon Mutation Based on Unbound Protein Structures

**DOI:** 10.3390/ijms26031343

**Published:** 2025-02-05

**Authors:** Zhengshan Chen, Song He, Xiangyang Chi, Xiaochen Bo

**Affiliations:** Academy of Military Medical Sciences, Beijing 100850, China; czs0076@163.com (Z.C.); or hesong@bmi.ac.cn (S.H.)

**Keywords:** antibody affinity changes, deep learning, antibody mutation, antigen–antibody complex, structure representation

## Abstract

Antibodies are key proteins in the immune system that can reversibly and non-covalently bind specifically to their corresponding antigens, forming antigen–antibody complexes. They play a crucial role in recognizing foreign or self-antigens during the adaptive immune response. Monoclonal antibodies have emerged as a promising class of biological macromolecule therapeutics with broad market prospects. In the process of antibody drug development, a key engineering challenge is to improve the affinity of candidate antibodies, without experimentally resolved structures of the antigen–antibody complexes as input for computer-aided predictive methods. In this work, we present an approach for predicting the effect of residue mutations on antibody affinity without the structures of the antigen–antibody complexes. The method involves the graph representation of proteins and utilizes a pre-trained encoder. The encoder captures the residue-level microenvironment of the target residue on the antibody along with the antigen context pre- and post-mutation. The encoder inherently possesses the potential to identify paratope residues. In addition, we curated a benchmark dataset specifically for mutations of the antibody. Compared to baseline methods based on complex structures and sequences, our approach achieves superior or comparable average accuracy on benchmark datasets. Additionally, we validate its advantage of not requiring antigen–antibody complex structures as input for predicting the effects of mutations in antibodies against SARS-CoV-2, influenza, and human cytomegalovirus. Our method shows its potential for identifying mutations that improve antibody affinity in practical antibody engineering applications.

## 1. Introduction

Antibodies are proteins that play an important role in the mammalian immune system, and the target molecules of antibodies, such as proteins or chemical ligands, are named antigens. Monoclonal antibodies (mAbs) are currently the largest class of bio-therapeutics in the clinic due to their high binding affinity and target specificity [1,2,3]. Antibody drug candidates often need to be engineered to improve affinity, specificity, stability, solubility and other properties. Improving affinity in particular is important for increasing drug efficacy and decreasing the amount of antibody per dose [4]. It is known that there is a distinction between the types of interactions used in antibody–antigen (Ab-Ag) binding and those observed in general protein–protein interactions [5]. Amino acid mutations can be introduced to existing antibodies to increase the binding affinity and specificity of the antibody [6], but there is no clear rule for identifying mutations that increase affinity. Affinity is experimentally measured with enzyme-linked immunosorbent assay (ELISA), surface plasmon resonance (SPR) or isothermal titration calorimetry (ITC). Constructing and expressing a large number of antibody mutants and measuring their affinity requires substantial time and cost. It makes sense to use the computational method that predicts the effect of antibody mutations on affinity before experimental evaluation.

A number of methods have also been developed to predict the changes in binding affinity in regard to mutation in recent years. These computational tools are largely divided into two categories, molecular energy-based approaches such as FoldX [7], EvoEF2 [8,9], Rosetta [10] and machine learning-based approaches such as mCSM toolkit [11,12,13,14,15], TopGBT [16], Hom-ML [17], GeoPPI [18], Geometric [19], BindFormer [20], GearBind [21]. All of the aforementioned methods require the 3D structures and the protein–protein complex in the bound state to predict changes in binding affinity upon mutation. However, an accurate complex structure, the prerequisite for ΔΔG prediction, is not easily available for most antibody–antigen pairs [21]. Furthermore, current multimer structure prediction methods, such as AlphaFold3 [22,23] and docking [24,25], are still insufficiently reliable as starting points for structure-based affinity maturation. While the performance of AlphaFold3 in predicting the structures of antibody–antigen complexes has improved compared to previous versions, it still lags behind the predictions for other complexes, exhibiting a 60% failure rate for antibody and nanobody docking when sampling a single seed [26].

To address the importance of predicting the change in affinity without an Ab-Ag complex structure, we have developed a deep learning-based framework, called MutAb, for predicting the effect of mutations on antibody affinity with learnable context-aware structural representations of antigens and antibodies. Given that the antigen–antibody complex structure is not a required input, MutAb exhibits more obvious advantages in antibody engineering applications compared to other competing methods.

## 2. Results

### 2.1. Overview

**Figure 1:**
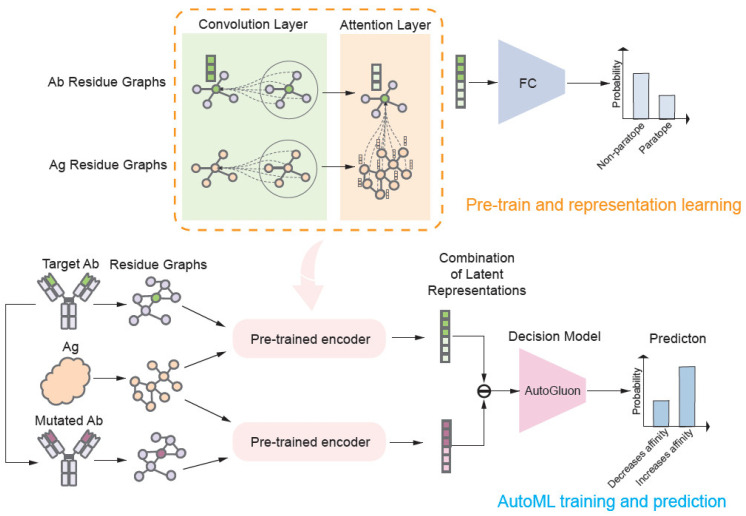
Overall framework. The structures of the wild-type antibody, mutant antibody and antigen are represented as residue-level graphs, respectively. These graphs are input into a pre-trained encoder (including convolution and attention modules) to generate residue-level representations of mutations in antibodies, which are then passed through the AutoGluon model to classify whether the mutations increase or decrease antibody affinity.

We propose a framework based on deep learning to predict the effect of mutations on antibody affinity without an antigen–antibody structure in the bound state. The learned representation module serves as an encoder in our framework to leverage biological insights (Figure 1).

To address the lack of a dedicated antibody mutation dataset, we curated a benchmark dataset containing 15 antibody cases and 424 single-point mutations entries, and evaluated our framework in the benchmark comparisons against predictors commonly used for this field. This comparison is not entirely fair to our framework because some of the predictors take the structure of an Ab-Ag complex in the bound state as input. Nonetheless, the evaluation demonstrates the outstanding or comparable performance of our framework. Furthermore, in predicting the effects of mutations on antibodies against SARS-CoV-2 (P36-5D2 and R3P1-E4), influenza (NC41 and NC10) and human cytomegalovirus (1G2), we demonstrated the advantages of our approach over energy-based methods and docking protocol, especially in scenarios where precision antigen–antibody complex structures are unavailable.

### 2.2. Benchmark Composition

**Figure 2:**
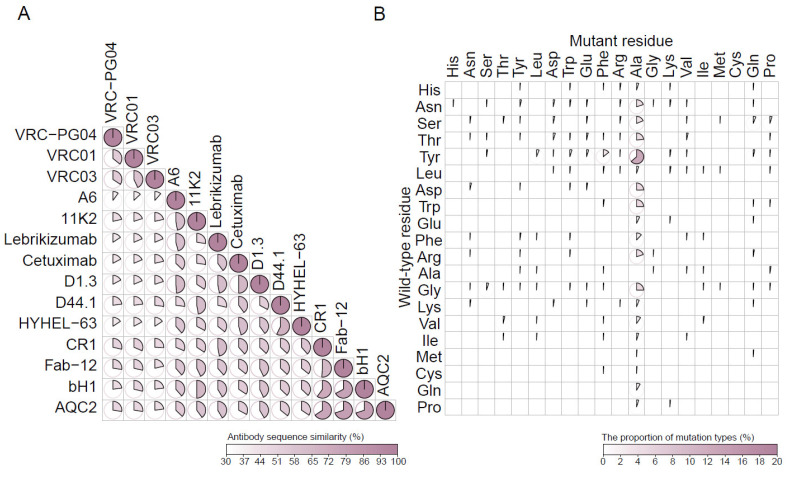
Antibodies and mutation types in the benchmark. (**A**) Pairwise similarity of antibody amino acid sequence. (**B**) Types of amino acids before and after mutation occurs.

The SKEMPI 2.0 dataset and several subsets (e.g., S645 [16], S1131 [27], S4169 [11], M1707 [28]) are widely used in ΔΔG prediction tasks. However, there is currently no subset that is strictly made up entirely of mutations on the antibody. Due to the asymmetry of the contact surfaces of antigens and antibodies, a pure antibody mutation dataset is necessary for the establishment of computer-aided antibody engineering methods. In order to develop our framework and benchmark against other methods, the benchmark of 15 unique antibodies was established and the maximum pairwise sequence similarity between antibodies was 79% (Figure 2A). We obtained the single-point mutation data on the antibody by filtering the SKEMPI 2.0 database and removed the mutations that could not be modeled by FoldX5 and EvoEF2. Specifically, we group mutations by unique antibodies, exclude groups with fewer than ten mutation data points and calculate metrics for each antibody group separately. As a result, our benchmark comprises 424 mutants across 15 structures of the Ab-Ag complex (Table 1). Most single-point mutations are located at the binding site, and the most common mutation is from tyrosine to alanine (i.e., 12.7%) (Figure 2B).

### 2.3. Representation Space for Antibody Mutations Generated by the Pre-Trained Encoder

A pre-trained scheme enforces encoder in MutAb to capture general rules in the amino acids type and position of the antibody residues that form paratope. Since the mutations lead to different composition and conformations in paratope, the pre-trained encoder is helpful in the prediction of the Ab-Ag binding affinity changes upon mutations. In the benchmark, we use the pre-trained encoder to generate representations for each mutation. Then, we employed the principal component analysis (PCA), the t-distributed stochastic neighbor embedding (t-SNE) and uniform manifold approximation and projection (UMAP) to compare the distribution of these representations in a low-dimensional space. These algorithms are widely used in machine learning to reduce the feature dimension and preserve the two most important components for the input representations.

**Figure 3:**
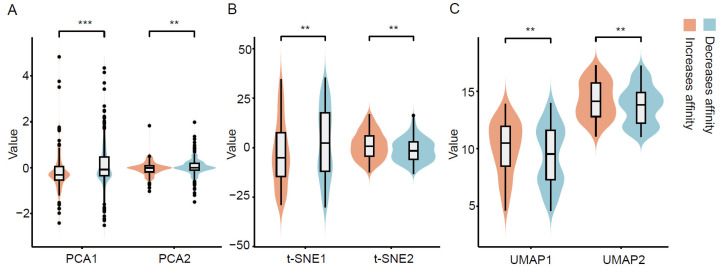
The mutation representation space after a dimensionality reduction. (**A**) The two most important components obtained by PCA. (**B**) The two dimensions of t-SNE across the groups. (**C**) The two dimensions of UMAP across the groups. Mutations in different groups are marked with different colors and significance is determined by an unpaired Wilcoxon test. ** *p* < 0.01, *** *p* < 0.001.

We show the low-dimensional representation space of mutations in the dataset (Figure 3). Additionally, we notice that between mutations increased affinity and mutations decreased affinity, and there are significant differences in both two preserved components of the mutation representations (*p* < 0.05). In other words, the representations produced by the pre-trained encoder have the potential to reflect the effect of mutations on antibody affinity.

### 2.4. Prediction of the Mutational Effects on Binding Affinity

We classified mutations into positive and negative effects based on the sign of ΔΔG. Based on the above mutation representation, we employed AutoGluon-Tabular (automated machine learning framework) to train and select the best machine learning model to predict classification labels. Given the imbalance and limited size of the dataset, we conducted a stratified three-fold cross-validation. Additionally, to more accurately reflect the real-world applications of predictive models, we implemented a leave-one-antibody-out cross-validation system. We calculated eight metrics, namely AUROC, AUPRC, ACC, BACC, F1 Score, precision, recall and MCC, which were commonly used in practical applications.

**Figure 4:**
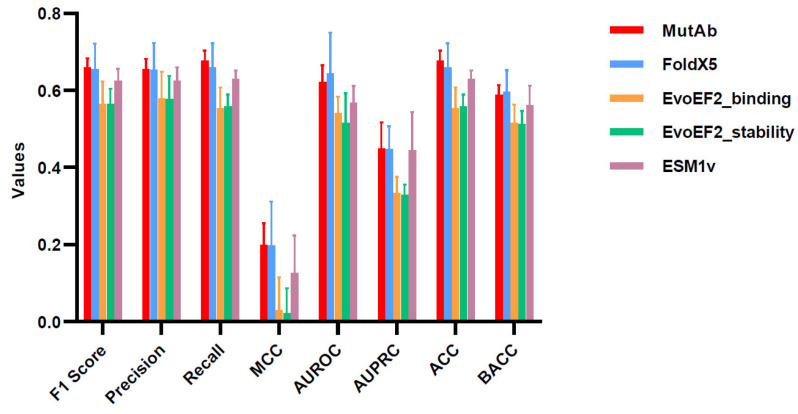
Evaluation of prediction in stratified three-fold cross-validation.

**Figure 5:**
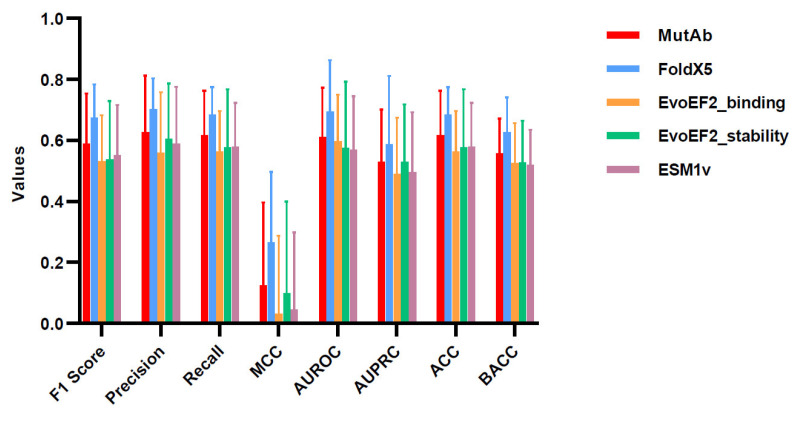
Evaluation of prediction in leave-one-antibody-out cross-validation.

The results for each baseline method are summarized in Figure 4 and Figure 5, while the corresponding *p*-values are presented in Tables S3 and S4. In both cross-validation methods, overall, the average performance of our framework exceeds that of the EvoEF2 tool and the ESM1v model but is slightly lower than that of the FoldX5 tool. The FoldX5 and EvoEF2 tools require an Ab-Ag complex structure in the bound state as input and use the energy function to assess the impact of residue mutations. Nonetheless, as we show in the next section, MutAb has a stronger robustness and wider applicability than the tools based on precise complex structures.

**Figure 6:**
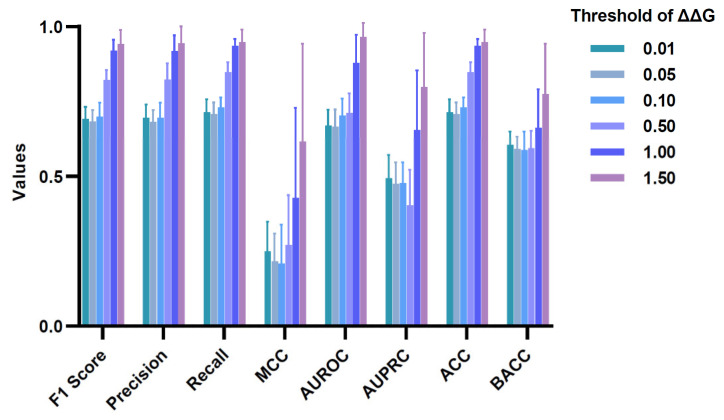
The performance of MutAb in predicting highly increasing affinity and highly decreasing affinity mutations.

We further evaluated the performance of MutAb to assess the quality of classifying mutations into highly increasing affinity mutations (ΔΔG > |Threshold|) and highly decreasing affinity mutations (ΔΔG < −|Threshold|). The performance evaluation metrics were calculated based on stratified three-fold cross-validation (randomly repeated five times), and different ΔΔG thresholds were tested. Figure 6 indicates the impressive performance of MutAb in predicting highly increasing affinity and highly decreasing affinity mutations, especially under the condition where the threshold is greater than 0.5 kcal/mol. A ΔΔG of the order ±0.5 kcal/mol is within the experimental error [29]. However, a considerable proportion of the ΔΔG entries (36.26%) in the dataset falls within the range of −0.5 to +0.5 kcal/mol, which makes classification challenging and limits the accuracy of predictions when no ΔΔG threshold is set.

### 2.5. Prediction of the Mutational Effects on Neutralization Against SARS-CoV-2 Pseudotyped Virus: Validation with a Blind Dataset

The severe acute respiratory syndrome coronavirus 2 (SARS-CoV-2) was used as one of the examples to test the realistic utility of our framework in antibody protein engineering. The entry of SARS-CoV-2 into host cells can be effectively blocked by antibodies, thus providing a promising therapeutic solution for the associated disease. Without an antibody against SARS-CoV-2 during the training phase of our framework, we tested whether MutAb can capture the effects of mutations in the antibodies on the neutralization of a SARS-CoV-2 pseudotyped virus. One of the definition of neutralization is “the reduction in viral infectivity by the binding of antibodies to the surface of viral particles (virions), thereby blocking a step in the viral replication cycle that precedes virally encoded transcription or synthesis” [30,31]. Compared with the Ab-Ag affinity, the neutralization activity of the antibody against a target virus is more reflective of the effect of the antibody in vivo in terms of protection or therapy. An enhanced Ab-Ag binding affinity is often correlated with increased neutralizing activity, as strong binding can prevent the antigen from interacting with its cellular receptors. The pseudovirus neutralization activity data of wild-type mAb (P36-5D2 and R3P1-E4) and single-point mutants was obtained from studies by Sisi Shan et al. [19] and Lili Li et al. [32]. The neutralization activity of wild-type mAbs and their single-point mutants against several strains of SARS-CoV-2 viruses was determined by a pseudovirus neutralizing assay. The neutralization curves of the antibody mutants were provided in the study by Lili Li et al. and the IC_50_ (half-maximal inhibitory concentration) values were provided in the study by Sisi Shan et al. For the specific strain of SARS-CoV-2 viruses, we calculate the log fold changes of IC_50_ for each mAb mutant relative to the wild-type mAb and use its sign as a classification label. Matched with different viral strains, all mutations of the two antibodies comprise a single dataset containing 135 entries.

**Figure 7:**
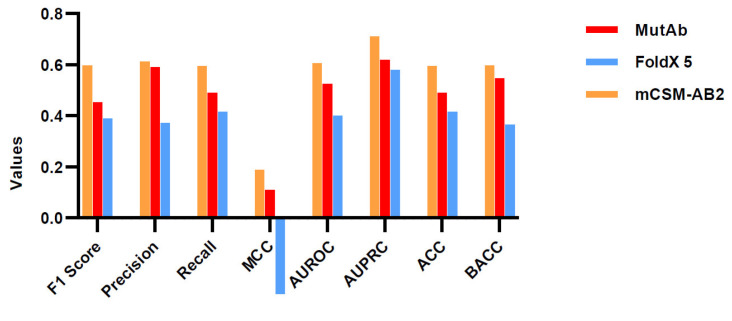
Evaluation of prediction in the anti-SARS-CoV-2 antibody datasets.

We focus on the method’s ability to classify mutations into two categories: mutations that enhance neutralizing activity and mutations that weaken neutralizing activity. Here, in addition to FoldX5, we introduce mCSM-AB2 as a comparison method. mCSM-AB2 is a graph-based machine learning approach that requires the input of the 3D structure of Ab-Ag complex to predict the effects of mutations on mAb binding affinity. The evaluation results are summarized in Figure 7. Interestingly, our framework achieved a better performance than that of the FoldX 5.

For most strains of SARS-CoV-2 viruses, there is no experimental Ab-Ag complex structure. Therefore, it is necessary to generate the residue mutation model structure from the initial complex structure as the input of FoldX and mCSM-AB2. Such an input condition makes the prediction task more difficult than it is for solved complexes structures for tools like FoldX. Not surprisingly, mCSM-AB2 achieves optimal performance. The mCSM-AB2 uses the complex structure in the bound state as input, while our framework only inputs the split structure in the unbound state. Additionally, the graph-based machine learning method is more resistant to reductions in the quality of the structure than the energy-based method.

### 2.6. Comparison with Docking Protocol

Antibody NC41 [33] and NC10 [34] binds to the subtype N9 neuraminidase (NA) of influenza virus and inhibits its enzyme activity. The 1G2 [35] antibody is a neutralizing human monoclonal antibody which binds to human cytomegalovirus (HCMV) glycoprotein B (gB) ectodomain. As a case study, we aim to assess whether our framework demonstrates superior predictive performance compared to the conventional molecular docking protocol for the three antibodies.

**Figure 8:**
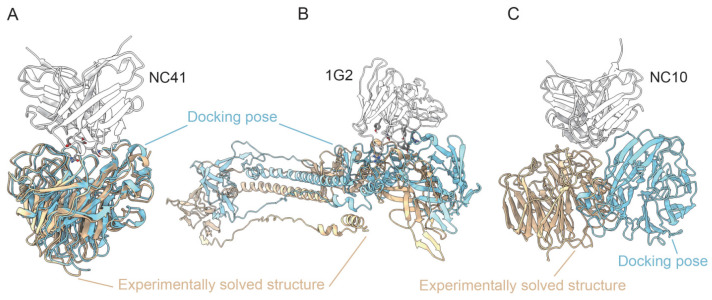
Ab-Ag docking and using the docking conformations as input for FoldX5 and mCSM-AB2. The optimal docking conformations for (**A**) NC41, (**B**) 1G2 and (**C**) NC10 obtained from ClusPro. The antibody component in the experimentally solved structure was superimposed with the antibody in the docking pose of the Ab-Ag complex. The antibody, the antigen in the experimentally solved structure and the antigen in the docking pose are represented in white, orange and cyan, respectively.

The structure of the antibody and antigen were separated from the experimentally resolved complex structure (PDB ID: 1NCA, 1NMB and 5C6T), and 10 docking conformations were generated using ClusPro’s Antibody Mode [36,37]. NC41 and 1G2 obtained the Ab-Ag docking conformation, which was close to the original natural structure through ClusPro (Figure 8A,B), while the docking conformation of NC10 was quite different from the natural structure (Figure 8C). The docking conformations ranked highest in ClusPro’s output (the center of the largest pose clusters) for NC41 and 1G2 were used as the input of FoldX and mCSM-AB2 to predict the effect of mutations on an antibody’s affinity.

**Figure 9:**
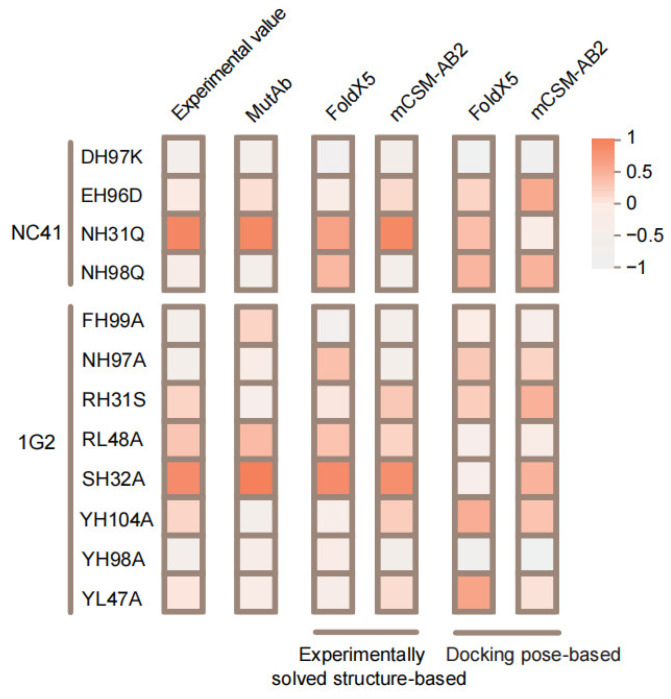
The heatmap shows the scaled values of experimental and predicted affinity changes. From left to right, it sequentially represents experimental ΔΔG, the probability of the mutation increasing antibody affinity predicted by our framework, predictive value from FoldX5 and mCSM-AB2 using the experimentally solved structure as input and the predictive value of FoldX5 and mCSM-AB2 using the docking pose as input.

When the experimentally solved structure was used as input, the predicted results of FoldX5 and mCSM-AB2 were consistent with the actual changes in the affinity of the antibody mutants. However, when using the docking conformation as input, the prediction performance of FoldX5 and mCSM-AB2 can be compromised, even resulting in outcomes that contradict those obtained by using an experimentally solved structure (Figure 9). When there is no experimentally solved antigen–antibody complex structure, our framework offers a distinct advantage. Even using a docking conformation that is similar to the natural structure as input for tools like Fold and mCSM-AB2 may lead to inaccurate predictions. Furthermore, the docking structure itself may not be reliable (such as NC10).

## 3. Discussion

Predicting mutations in the antibody that are beneficial to its function is a key challenge in helping to guide the maturation of conventional affinity. Although some successful computationally guided Ab development examples have been published in recent years, the computational tools have not yet had a broad transformative impact on antibody engineering due to the limited scope of application of the available methods or the high requirements for input data, such as the need for accurate antigen–antibody complex structures. In this study, we present a deep learning framework, MutAb, that utilizes a pre-trained model as an encoder for the prediction of beneficial residue mutations on antibody. In cross-validation using the benchmark dataset, our results show that the framework is able to distinguish the mutations in antibodies that increased the Ab-Ag affinity and decreased the Ab-Ag affinity across different antibodies. This also highlights the power of the pre-trained model as an encoder in terms of identifying the contribution of residues on the paratope by efficiently representing the intramolecular and intermolecular structural environment of target residues.

We applied MutAb to predict the impact of mutation on the neutralization activity of mAb (P36-5D2 and R3P1-E4) against different strains of SARS-CoV-2 viruses. The receptor-binding domain (RBD) of the spike protein of different SARS-CoV-2 strains differs by only one to five amino acids. Using the complex structure of the wild-type spike RBD and antibody as a homology reference, we simulated the complex structures of the RBDs of the different strains in combination with the antibody. When FoldX and mCSM-AB2 use the simulated antigen structure as input, the prediction accuracy of our method surpasses that of FoldX, coming in second only to mCSM-AB2. The mCSM-AB2 utilizes graph-based structural signatures and machine learning techniques, enabling it to maintain a robust performance even when utilizing homology models as input.

In more common scenarios, where no existing antigen–antibody complex structure can serve as a homology reference, it is necessary to use docking methods to generate an Ab-Ag complex conformation before applying tools such as FoldX and mCSM-AB2. Under these conditions, MutAb demonstrates a distinct advantage. For the NC41, NC10, and 1G2 antibodies, we compared our approach against the docking process. When FoldX and mCSM-AB2 utilize the docking pose as input, our framework has a better prediction performance. The discrepancy between the docking conformation and the actual natural structure significantly affects the prediction outcomes of both FoldX and mCSM-AB2. For the external validation set of P36-5D2 and R3P1-E4 (mAbs against SARS-CoV-2), in the case studies of NC41, NC10 (mAbs against influenza) and 1G2 (mAb against human cytomegalovirus), the antibodies and antigens involved are both unseen in the training and test datasets. The results demonstrate the generalization ability and usefulness of this method.

We observed that, during cross-validation, the model performs much better on the training dataset compared to the test dataset (Table S2). Given the limited sample size, there is a possibility that overfitting occurred during the training process. However, the distinct mutation entities present in each fold of the cross-validation, along with the unseen data in the external validation set, help mitigate the risk of overestimating the model’s performance due to overfitting. The performance of our model also indicates that it has captured valuable information, thereby enhancing our understanding of the underlying patterns in antibody mutation data. Though our framework is demonstrated to have advantages over other competing methods, it also has some limitations. The binding affinity of the antibody to the target antigen is determined by the whole interaction surface, and the representations of antibody mutations is limited to a certain residue and its neighbor residues. Therefore, our framework is better suited for classification tasks, such as predicting whether a mutation in the antibody is advantageous or disadvantageous for Ab-Ag binding, rather than directly predicting the ΔΔG value. Another issue is that our method requires the generation of sequence profiles (especially PSSM), which takes an average of 8.14 ± 3.36 min per structure across all the datasets used in this work. Therefore, our method is more suitable for directed mutation optimization at several known key residue sites (e.g., as determined by alanine scanning mutation experiments) for a monoclonal antibody rather than for the prediction of high-throughput deep mutation scans.

## 4. Materials and Methods

### 4.1. Definition of the Task of Predicting Antibody–Antigen Binding Affinity Changes upon Mutations on Antibodies

Given the interaction of the antibody–antigen, the residue on the antibody to be mutated to the new amino acid type, the goal is to estimate the affinity or binding free energy change between the original antibody–antigen and mutant.

(1) $$\Delta \Delta G=\Delta G_{\mathrm{wild}-\mathrm{type}}-\Delta G_{\mathrm{mutant}}$$

where

(2) $$\Delta G=RT\cdot \ln K_{dissociated}$$

In the equation, *R*, *T* and *K* are the gas constant, temperature in Kelvin, and dissociation (or inhibition) constant (i,e., $K_{dissociated}$ or $K_{inhibition}$), respectively. The dissociation constant $K_{dissociated}$ reflects the affinity of the antibody to the target antigen, with smaller values and a stronger affinity. The positive value of ΔΔG indicates a stronger binding affinity between the antibody mutant and antigen, which corresponds to a smaller $K_{dissociated}$ value for the antibody mutant. Additionally, the negative value of ΔΔG indicates a weaker binding affinity. The binary classification task is to predict whether the value of ΔΔG is negative or non-negative.

### 4.2. Data and Preprocessing

To develop our predictive pipeline, we collected the ΔΔG data of single-point mutations from the mCSM-AB2 dataset (a subset of SKEMPI 2.0) and downloaded the experimentally determined 3D structures complexes from the SKEMPI2.0 databases. Compared with the mCSM-AB2 dataset, we discarded nanobodies, anti-idiotype antibodies, antibodies with fewer than 10 mutations, mutations not present on the antibodies and mutations that could not be simulated by FoldX5 or EvoEF2. This resulted in a benchmark dataset that consists of 15 unique antibodies and 424 mutation entries. A total of 135 entries with a ΔΔG value of 0 or greater were classified as affinity-increasing mutations, while 289 entries with a ΔΔG value less than 0 were classified as affinity-reducing mutations.

As for the single-point mutation dataset for neutralizing antibodies targeting SARS-CoV-2 spike protein, we collected 2 mAbs (P36-5D2 and R3P1-E4), each complexed with SARS-CoV-2 spike RBD separately. The structures of these mAbs are available (PDB ID: 7FAF and 7VMU). The effects of mutations on these two antibodies on neutralization against SARS-CoV-2 pseudotyped virus were measured by Sisi Shan et al. [19] and Lili Li et al. [32]. We included qualitative and quantitative assay data, leading to a total of 135 data points for the classification task. Using EvoEF2, the structures of the spike RBD from different strains were generated by substituting amino acids in the WT structure and subsequently optimized.

We used BIOVIA Discovery Studio 4.5 (Dassault Systèmes BIOVIA, San Diego, CA, USA) to extract the 3D structures of antigens and antibodies from the complexes, respectively. The antibody Fv sequence was annotated by the “Annotate Antibody Sequence” tool in the BIOVIA Discovery Studio. Only the Fv region was retained for each antibody structure. The antibody mutant structures were generated using EvoEF2, which also optimized the rotamers of the mutated and surrounding residues.

### 4.3. Constructing the Graph Structure of Antibody and Antigen

To construct a graph representing the structure of a given antibody or antigen, we treated the individual residues as nodes and the Cβ-Cβ distances between them as the edges. For glycine (Gly) residues, which lack a β-carbon, we utilized the Cα atom instead. Edges with a distance of less than 10 Å are retained. We initialized the embeddings for each node by incorporating both structural and sequence features at the residue level from the unbound structure of the antibody or antigen. The structural features are composed of the solvent accessible surface area (ASA) and a half-sphere amino acid composition (HSAAC). The absolute and relative solvent accessible surface areas of the residue (d = 2) were calculated by STRIDE [38]. As defined in PAIRpred [39], HSAAC (d = 20) is a local amino acid profile that indicates the frequency of each amino acid type within 8 Å of the target residue. Sequence-based features comprise the amino acid type represented by one-hot encoding (d = 20) and evolutionary information derived from the position-specific scoring matrix (PSSM). The PSSM (d = 20) is generated by employing the PSI-BLAST [40] to query the NCBI’s Non-Redundant sequence database (https://ftp.ncbi.nlm.nih.gov/blast/db/FASTA/nr.gz, accessed on 5 October 2023). The PSSM encodes each residue as a vector with 20 elements, representing the probabilities of the 20 amino acids occurring at that position. The effectiveness of PSSM in predicting protein–protein interaction (PPI) sites has been demonstrated [41]. We have modified the myPDB.py Python script from PAIRPred to compute the total of the 62 dimensional features mentioned above.

### 4.4. Implementation Details of the Representation Learning Module

The representation learning module comprises a graph convolution layer and a attention layer and shares the basic idea of the PECAN framework [42]. The graph convolution implemented by Fout et al. [43] enables order-independent aggregation of properties across a neighborhood of antibody/antigen residues that collectively contributes to the formation of an Ab-Ag binding interface. To summarize, considering that $x_{i}$ is the initial representation of the target node *i* in the antibody’s residue graph and $N_{i}=\{x_{n_{1}},\ldots ,x_{n_{k}}\}$($i\notin \{n_{1},\ldots ,n_{k}\}$) is the representation set of neighbor nodes that define the receptive field of the convolution, the aggregate weight matrix of the target node (i.e., center node) is $W^{c}$ and the aggregate weight matrix of the neighbor node is $W^{n}$. After the convolution operation, the representation of the target node is updated to

(3) $$x_{i}^{\prime }=\mathrm{ReLU}\left(W^{c}x_{i}+\frac{1}{\left|N_{i}\right|}\sum_{j\in N_{i}}W^{n}x_{j}+b\right)$$

An attention layer, designed by Luong T et al. [44] and Pittala S et al. [42] encodes the contextual representations of the antigen’s residue graph onto the antibody’s residue graph, providing potential information about the interaction surface residues on the antigen for the target residues on the antibody. Considering that $z_{m}$ is the initial representation of node m in the antigen’s residue graph, similarly, after the convolution operation, the representation of the node is updated to $z_{m}^{\prime }$. The attention score between antibody node *i* and antigen node *m* is expressed as $\mathrm{ReLU}\left({\left(W^{a}x_{i}^{\prime }\right)}^{T}(W^{a}z_{m}^{\prime })\right)$. By accumulating the product of all antigen node representations and their corresponding normalized attention scores, the antigen context representations for the antibody’s residue graph node *i* is obtained. The combination of $x_{i}$ and context forms the representations of the target residue on the antibody, thus realizing the functionality of the encoder in our framework.

In order to determine the learnable parameters $W^{c}$, $W^{n}$, $W^{a}$ in the representation generation module of our framework, we retrained the paratope prediction model in the PECAN framework, incorporating a fully connected layer for classification, as described in the original paper. Additionally, the training and test data utilized are from 460 Ab-Ag complexes (https://zenodo.org/records/3885236, accessed on 27 March 2024). Such a pre-trained scheme enables our representation generation module to capture general rules regarding the amino acid types and the positions of the antibody residues that form the paratope.

### 4.5. Model Training and Performance Evaluation Metrics

The pre-trained module encodes the wild-type and mutant residues on the antibody, and the difference between the two was fed into the AutoGluon-Tabular [45], an open-source AutoML framework used to train the model and predict changes in antibody affinity. A stratified three-fold cross-validation approach was employed on the benchmark dataset using the StratifiedKFold function from the sklearn.model_selection Python module. With this approach, the dataset was divided into three folds, with each fold containing distinct mutation entities not present in the others while preserving the class ratio in each fold to match that of the entire dataset. Two of the folds were utilized for training purposes, while the third fold was reserved for testing. Stratified k-fold cross-validation is an extension of regular k-fold cross-validation that is particularly useful for handling imbalanced and small-sized datasets.

Additionally, a leave-one-antibody-out cross-validation system was also employed. Mutation entries from 14 out of 15 antibodies were used for training, while the mutation entries from the remaining antibody served as the test set. Since each antibody in the benchmark dataset is unique, this approach rigorously evaluates the model’s generalization.

The classification performance of the model was evaluated by calculating accuracy (ACC), balanced accuracy (BACC), the area under the precision–recall curve (AUCPR), the area under the receiver operating characteristic curve (AUCROC), the F1 score, precision, recall, and the Matthews correlation coefficient (MCC) using the sklearn.metrics Python library.

### 4.6. FoldX5, EvoEF2 and ESM1v

FoldX [7] provides a quantitative analysis of the impact of mutations on the stability, folding, and dynamics of proteins and protein complexes (http://foldxsuite.crg.es, accessed on 20 October 2023). We undertook a comparison with the new version of FoldX, FoldX5. EvoEF2 [8] is an accurate energy function for protein sequence design, protein energy computing, and building mutant models (https://github.com/tommyhuangthu/EvoEF2, accessed on 20 October 2023). The ComputeStability command in EvoEF2 computes the stability (total energy) of the protein complex. The ComputeBinding command in EvoEF2 calculates the binding interaction energy of a protein–protein complex, dividing the designation of the chains into two components: antigen and antibody. The two are referred to here as EvoEF2_stability and EvoEF2_binding, respectively. ESM1v [46] is a protein language model designed for predicting the effects of variants (https://github.com/facebookresearch/esm/, accessed on 27 June 2023). Since the ESM1v models only accept a single sequence as input, we concatenated the sequences of the antibody and antigen proteins for the analysis.

### 4.7. Antibody–Antigen Docking

ClusPro [36] employs shape complementarity, electrostatics, and desolvation energy terms to generate the multiple docking poses of the protein complex. ClusPro outputs the docking poses at the centers of the 10 most populated clusters and ranks the models by cluster size. The ClusPro web server (https://cluspro.org, accessed on 11 August 2024) was used in Antibody Mode with default settings, providing automated masking for the non-CDR regions of antibodies. The quality of protein interfaces in the docking poses was assessed against the experimentally solved structures using DockQ [47] (https://github.com/bjornwallner/DockQ, accessed on 11 August 2024).

## 5. Conclusions

Ab-ag complex crystal structures will only be available for a tiny number of the antibodies of interest [48,49]. Due to the flexibility of complementarity-determining regions and the absence of co-evolution signals, Ab-Ag complex modeling has been a long-standing challenge [25]. It is essential for structure-based ΔΔG predictors to work on antibody and antigen structures in the unbound state. In this study, we developed a deep learning-based framework for antibody affinity maturation. This framework utilizes a pre-trainable Ab-Ag context-aware structural representation encoder and does not require the structure of the Ag-Ab complex as input. We have demonstrated that this framework is practical in antibody engineering and has the potential power to improve the efficiency of antibody development.

## Figures and Tables

**Table 1:** Characterization of antibodies and the number of mutations for each subset.

Name	PDB ID	Organism	Antigen	Mutations Number
HYHEL-63	1DQJ, 1XGP, 1XGQ, 1XGR, 1XGT, 1XGU, 3HFM	Mus musculus	Hen egg white lysozyme	61
Herceptin/bH1 anti HER2	1N8Z, 3BE1	Chimeric/Homo sapiens	Human Epidermal GrowthFactor Receptor 2 (HER2)	58
AQC2	1MHP, 2B2X	Mus musculus	Integrin alpha-1	53
VRC01	3NGB	Homo sapiens	HIV-1 gp120	41
D1.3	1KIP, 1KIQ, 1KIR, 1VFB	Mus musculus	Hen egg white lysozyme	37
bH1 anti VEGF	3BDY	Homo sapiens	Vascular endothelial growth factor (VEGF)	29
VRC03	3SE8	Homo sapiens	HIV-1 gp120	28
VRC-PG04	3SE9	Homo sapiens	HIV-1 gp120	25
A6	1JRH	Mus musculus	Interferon gamma receptor (IFNgammaR) alpha-chain	18
Cetuximab	1YY9	Chimeric	Epidermal growth factor receptor	16
11K2	2BDN	Mus musculus	Monocyte chemoattractant proteins (MCPs)	12
CR1	2NYY	Homo sapiens	Botulinum neurotoxin type A	12
Lebrikizumab	4I77	Homo sapiens	Interleukin-13 (IL13)	12
D44.1	1MLC	Mus musculus	Hen egg white lysozyme	11
Fab-12	1BJ1, 1CZ8	Mus musculus	Vascular endothelial growth factor (VEGF)	11

## Data Availability

The datasets and code used in this work are available at https://github.com/shanshangg/MutAb, accessed on 3 January 2025.

## Supplementary Materials

The following supporting information can be downloaded at: https://www.mdpi.com/article/10.3390/ijms26031343/s1.
