# Supplementary material for: Predicting Antibody Affinity Changes upon Mutation Based on Unbound Protein Structures
Source: Int J Mol Sci. 2025 Feb 5;26(3):1343. doi: 10.3390/ijms26031343 (PMC11818220; doi:10.3390/ijms26031343)
Supplement: Supplementary file 1 [file ijms-26-01343-s001.zip › ijms-3441983-supplementary.pdf]

Supporting Information

Predicting Antibody Affinity Changes upon Mutation Based on Unbound Protein Structures

Additional Results

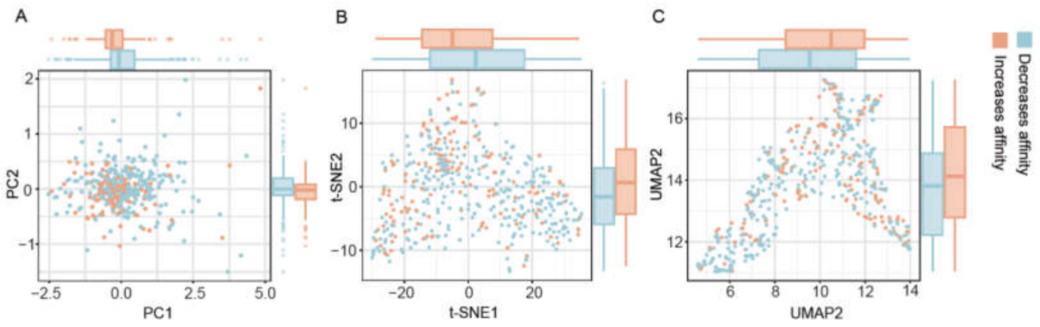

Figure S1. Visualization of the mutation representation space. (A) The two most important components obtained by PCA. (B) The two dimensions of t-SNE across the groups. (C) The two dimensions of UMAP across the groups. Orange indicates mutations that increase binding affinity, while cyan represents mutations that decrease binding affinity.

Table S1. Summary of performance in paratope prediction (pre-training task)

Data split	# Ab-Ag complex	# residues (# paratope)	AUPRC
Training	203	50350 (4389)	0.703±0.00339
Test	152	35373 (3311)	0.692±0.00288
Validation	103	25236 (2236)	0.711±0.00327

Table S2. Evaluation metrics used for the training set in cross-validation of the MutAb

Cross-Validation	AUROC	AUPRC	ACC	BACC	F1 Score	Precision	Recall	MCC
Stratified 3-fold cross-validation	0.970± 0.013	0.948± 0.029	0.956± 0.002	0.944± 0.002	0.956± 0.002	0.957± 0.002	0.956± 0.002	0.899± 0.004
Leave-one-antibody-out cross-validation	0.950± 0.021	0.919± 0.044	0.922± 0.033	0.903± 0.040	0.921± 0.033	0.923± 0.032	0.922± 0.033	0.822± 0.074

Table S3. *P*-values for prediction performance comparison in stratified 3-fold cross-validation

Method Comparison	AUROC	AUPRC	ACC	BACC	F1 Score	Precision	Recall	MCC
FoldX5 vs. MutAb	0.386	0.52	0.647	0.42	0.539	0.519	0.647	0.506
EvoEF2_binding vs. MutAb	**0.039**	**0.039**	**0.02**	**0.047**	**0.045**	0.096	**0.02**	**0.027**
EvoEF2_stability vs. MutAb	0.06	**0.039**	**0.004**	**0.02**	**0.016**	0.07	**0.004**	**0.012**
ESM1v vs. MutAb	0.098	0.474	**0.039**	0.23	0.1	0.15	**0.039**	0.168

P-value from the t-test comparing the performance evaluation metrics of MutAb with that of the control method. *P*-values less than 0.05 are highlighted in **bold**.

Table S4. *P*-values for prediction performance comparison in leave-one-antibody-out cross-validation

Method Comparison	AUROC	AUPRC	ACC	BACC	F1 Score	Precision	Recall	MCC
FoldX5 vs. MutAb	0.089	0.219	0.076	**0.048**	0.053	0.09	0.076	0.069
EvoEF2_binding vs. MutAb	0.403	0.273	0.147	0.252	0.163	0.176	0.147	0.169
EvoEF2_stability vs. MutAb	0.312	0.492	0.258	0.268	0.212	0.381	0.258	0.405
ESM1v vs. MutAb	0.249	0.306	0.236	0.19	0.264	0.297	0.236	0.208

P-value from the t-test comparing the performance evaluation metrics of MutAb with that of the control method. *P*-values less than 0.05 are highlighted in **bold**.

Table S5. Results of ClusPro 2.0 Antibody Mode

Antibody name	Docking pose	Cluster size	Weighted Scores	DockQ	irms	Lrms	fnat
NC41	model.000.00	100	-408.0	0.891	0.809	1.275	0.921
NC41	model.000.01	83	-310.6	0.012	15.572	52.507	0.000
NC41	model.000.02	47	-282.9	0.007	19.837	69.738	0.000
NC41	model.000.03	45	-272.9	0.011	17.950	50.643	0.000
NC41	model.000.04	44	-262.8	0.017	11.346	45.555	0.000
NC41	model.000.05	39	-286.0	0.010	17.062	55.985	0.000
NC41	model.000.06	36	-257.0	0.008	17.696	66.974	0.000
NC41	model.000.07	33	-270.9	0.007	18.035	68.152	0.000
NC41	model.000.08	30	-262.6	0.031	15.895	48.095	0.053
NC41	model.000.09	30	-251.0	0.034	9.916	31.740	0.013
1G2	model.000.00	84	-349.2	0.057	7.236	21.918	0.000
1G2	model.000.01	44	-386.1	0.004	27.852	86.553	0.000
1G2	model.000.02	41	-336.0	0.014	14.911	47.796	0.000
1G2	model.000.03	41	-362.6	0.005	20.504	83.544	0.000
1G2	model.000.04	35	-308.6	0.006	20.089	79.430	0.000
1G2	model.000.05	34	-311.6	0.066	7.435	23.455	0.042
1G2	model.000.06	31	-354.3	0.007	17.971	74.777	0.000
1G2	model.000.07	30	-341.6	0.004	30.639	92.879	0.000
1G2	model.000.08	29	-336.7	0.008	16.599	67.943	0.000
1G2	model.000.09	28	-390.7	0.014	12.246	50.876	0.000
NC10	model.000.00	89	-318.5	0.012	15.174	51.668	0.000

NC10	model.000.01	76	-284.1	0.010	19.711	55.679	0.000
NC10	model.000.02	39	-362.8	0.015	14.894	43.750	0.000
NC10	model.000.03	38	-301.4	0.005	22.314	79.521	0.000
NC10	model.000.04	37	-280.6	0.005	20.531	85.348	0.000
NC10	model.000.05	32	-288.3	0.015	11.230	51.422	0.000
NC10	model.000.06	32	-321.7	0.024	14.152	41.062	0.021
NC10	model.000.07	31	-335.5	0.020	13.374	37.656	0.000
NC10	model.000.08	30	-281.1	0.059	8.119	41.363	0.104
NC10	model.000.09	26	-295.9	0.050	13.696	29.800	0.063

The DockQ, irms, Lrms and fnat metrics are calculated using a Python tool called DockQ[1] (https://github.com/bjornwallner/DockQ).

Table S6. Experimental and predicted affinity changes of NC41 and 1G2

**Antibody name**	**Mutation**	**Exp. ΔΔG**	**MutAb probit**	**FoldX5#**	**FoldX5***	**mCSM-AB2#**	**mCSM-AB2***
NC41	DH97K	-0.71	0.819	-5.195	-9.492	-1.660	-1.900
NC41	EH96D	-0.41	0.508	-2.960	-2.222	-0.870	-0.470
NC41	NH31Q	0	0.103	-0.395	-1.037	0.010	-1.190
NC41	NH98Q	-0.54	0.788	-1.001	-0.581	-1.560	-0.570
1G2	FH99A	-1.46	0.778	-3.101	0.045	-2.920	-4.000
1G2	NH97A	-1.53	0.889	0.306	1.130	-2.930	-1.460
1G2	RH31S	-0.31	0.976	-0.666	1.001	-0.600	-0.240
1G2	RL48A	-0.12	0.707	0.218	-1.013	-0.890	-2.920
1G2	SH32A	0.87	0.517	1.918	-0.930	0.940	-0.310
1G2	YH104A	-0.36	1.000	-1.787	1.869	-0.720	-0.890
1G2	YH98A	-1.72	0.939	-1.327	-2.167	-3.110	-6.420
1G2	YL47A	-0.55	0.892	-1.723	2.117	-1.120	-1.950

# Experimentally solved structure-based.

* Docking pose-based.

REFERENCES

(1) Mirabello, C.; Wallner, B. DockQ v2: improved automatic quality measure for protein multimers, nucleic acids, and small molecules. *Bioinformatics* **2024**, *40* (10), btae586.
